# Disease Burden Of Co-Occurring Borderline Personality Disorder In Patients With Bipolar Disorder

**DOI:** 10.1192/j.eurpsy.2022.1166

**Published:** 2022-09-01

**Authors:** M. Turki, M. Abdellatif, N. Gargouri, S. Ellouze, S. Blanji, A. Daoud, N. Halouani, J. Aloulou

**Affiliations:** Hedi Chaker University Hospital, Psychiatry “b” Department, Sfax, Tunisia

**Keywords:** comorbidity, bipolar disorder, borderline personality disorder

## Abstract

**Introduction:**

In recent years, advances in the areas of both bipolar disorder (BD) and borderline personality disorder (BPD) have generated considerable interest in the relationship between these two conditions, since that they are commonly comorbid.

**Objectives:**

We aimed to investigate the impact of BPD on course of illness in patients with BP.

**Methods:**

We conducted a cross-sectional, descriptive and analytical study among 30 psychiatric outpatients diagnosed with BD in the Psychiatry « B » department, Hedi Chaker Hospital (Sfax, Tunisia). The McLean Screening Instrument for Borderline Personality Disorder (MSI-BPD) was used to screen for BPD. Clinical outcomes (hospital stays, comorbidities, suicidality…) were compared between BD- patients with or without BPD comorbidity.

**Results:**

The mean age was 41.63 years, with a sex ratio of ½. Among the patients, 2/3 were diagnosed with BD-I, while 1/3 presented a BD-II. Physical comorbidities, comorbid anxious and eating disorders were noted respectively in 36.7%; 16.7% and 43.3% of patients. Suicidal attempts were reported in 46.7% of cases. According to MSI-BPD, a comorbid BPD was noted in 30% of our sample. Patients with BD-II were significantly more likely to present BDP traits (50%) than those with BD-I (20%) (p<0.001). Patients with BPD were significantly more likely to attempt suicide (p=0.033), and to present physical comorbidities (p<0.001) and comorbid eating disorders (p<0.001).

**Conclusions:**

Our study showed that BPD darkens the prognosis of BD, because of worse outcomes related to suicide, physical and psychiatric comorbidities. Thus, its co-occurrence complicates the management of BD.

**Disclosure:**

No significant relationships.

